# AI-assisted task-based language learning and English proficiency: a dual-pathway model of cognitive engagement and self-regulated learning with AI trust as a boundary condition

**DOI:** 10.3389/fpsyg.2026.1842773

**Published:** 2026-06-15

**Authors:** Juan Liu, Jie Zhou

**Affiliations:** 1 School of Humanities and Law, Nanchang Jiaotong Institute, Nangchang, China; 2 General Office, Jiangxi Education Evaluation and Assessment Institute, Nanchang, China

**Keywords:** AI trust, AI-assisted learning, cognitive engagement, English proficiency, self-regulated learning, task-based language learning

## Abstract

**Purpose:**

Artificial intelligence is increasingly transforming language learning environments, yet the psychological mechanisms through which AI-assisted instructional practices influence language outcomes remain insufficiently understood. Although prior studies have examined AI-supported learning, limited research has explained how AI-assisted task-based learning activates distinct cognitive and behavioral processes that contribute to English proficiency in Chinese higher education contexts. Therefore, this study examines how AI-assisted task-based language learning influences English proficiency among university students in China by integrating cognitive engagement and self-regulated learning as parallel mediating mechanisms, with AI trust functioning as a moderating boundary condition.

**Methods:**

Data were collected from 412 undergraduate students enrolled in Chinese universities and analyzed using partial least squares structural equation modeling.

**Results:**

The results reveal that AI-assisted task-based learning significantly enhances both cognitive engagement and self-regulated learning, which subsequently improve English proficiency. Notably, self-regulated learning demonstrated substantially stronger direct and mediating effects than cognitive engagement, suggesting that AI-assisted learning environments may operate more effectively through behavioral regulation processes. Furthermore, AI trust strengthens the positive influence of AI-assisted learning on English proficiency.

**Implications:**

By integrating instructional design, learner cognitive processes, and trust in artificial intelligence within a unified analytical framework, the study advances a mechanism-based explanation of AI-enabled language learning and highlights how AI technologies can enhance educational effectiveness in higher education language learning environments.

## Introduction

Rapid technological developments are reshaping educational environments, particularly through the integration of artificial intelligence into learning systems. Universities increasingly adopt AI-supported tools such as intelligent tutoring systems, automated feedback platforms, and adaptive learning technologies to enhance students’ learning experiences (Poli and Morgana, 2025). Within language education, these developments have created new opportunities for interactive and personalized learning. Task-based language teaching has long been recognized as an effective instructional approach because it engages learners in meaningful communication and authentic language use (Simatupang and Heryono, 2026). When AI tools are integrated into such environments, learners benefit from continuous feedback and adaptive support, which facilitate language development (Huang et al., 2024; Feng, 2025).

Recent studies have increasingly demonstrated that AI-supported learning environments can positively contribute to language development and English proficiency outcomes across higher education contexts (Huang et al., 2024; Wang et al., 2025). In Chinese higher education institutions, English proficiency is particularly important because it influences students’ academic performance, access to international educational resources, research communication, and future professional competitiveness (Li et al., 2024; Zhang Q. et al., 2025; Zhang Z. et al., 2025). However, while prior research has largely established the effectiveness of AI-assisted language learning, existing studies have primarily emphasized outcome-oriented relationships and have provided comparatively limited explanation regarding the distinct psychological mechanisms through which AI-assisted task-based learning environments translate into improved English proficiency. Specifically, insufficient attention has been devoted to understanding how AI-supported instructional environments simultaneously activate learners’ cognitive engagement and self-regulated learning processes as complementary pathways influencing language outcomes.

Cognitive engagement represents a key mechanism linking instructional practices with learning outcomes. It reflects learners’ effort, persistence, and strategic thinking during learning activities (Feng, 2025). In language learning contexts, engaged learners process linguistic information more actively and demonstrate stronger performance (Huang et al., 2024). Technology-supported environments further stimulate engagement through interactive tasks and feedback (Feng, 2025). In AI-assisted contexts, engagement becomes more cognitively demanding, as learners must actively interpret, evaluate, and apply AI-generated feedback, thereby deepening their processing of language tasks (Feng, 2025; Huang et al., 2024). However, empirical evidence on how AI-assisted task-based learning enhances cognitive engagement remains limited.

Self-regulated learning is another critical determinant of language learning outcomes. Successful learners regulate their cognitive and motivational processes through goal setting, monitoring, and strategy adjustment (Zhang Q. et al., 2025; Zhang Z. et al., 2025). Learners who manage their learning behaviors effectively tend to achieve stronger academic outcomes (Lin, 2021). In language learning, self-regulation enables learners to practice independently and adapt strategies to overcome difficulties. Despite the potential of digital tools, limited research has examined how AI-assisted task-based environments foster self-regulated learning. This is particularly important because AI-supported feedback and adaptive learning features may only improve proficiency when learners actively monitor and regulate their learning strategies (Zhang Q. et al., 2025; Zhang Z. et al., 2025; Lin, 2021). Thus, understanding how AI-assisted environments simultaneously stimulate both cognitive engagement and self-regulated learning may provide a more comprehensive explanation of how AI-supported instructional practices influence language proficiency.

Beyond cognitive and behavioral mechanisms, learners’ perceptions of technology also shape learning effectiveness. Trust influences individuals’ willingness to rely on technological systems and accept automated recommendations (Akman Yeşilel, 2025). In human–AI interaction, trust determines how individuals collaborate with and utilize AI systems (Nazaretsky et al., 2022). Learners who perceive AI tools as reliable are more likely to use AI-generated feedback in their learning processes. However, empirical evidence on the moderating role of AI trust in AI-assisted language learning remains scarce. In particular, it remains unclear whether trust strengthens the extent to which AI-assisted learning experiences translate into improved English proficiency (Akman Yeşilel, 2025; Wang et al., 2025).

Taken together, these perspectives suggest that AI-assisted task-based language learning may not influence language outcomes through a single linear mechanism, but rather through multiple interrelated psychological processes. Specifically, instructional environments supported by artificial intelligence may simultaneously activate learners’ cognitive engagement and self-regulated learning, reflecting two complementary yet distinct pathways of learning behavior (Zhang Q. et al., 2025; Zhang Z. et al., 2025). While cognitive engagement captures learners’ depth of information processing, self-regulated learning reflects their ability to manage and adapt their learning activities over time (Lin, 2021). Importantly, the effectiveness of these processes may further depend on learners’ trust in AI systems, which shapes the extent to which they rely on and utilize AI-generated feedback (Nazaretsky et al., 2022). Despite these theoretical connections, prior research has rarely integrated these mechanisms within a unified framework, particularly in the context of AI-assisted task-based language learning.

The present study examines how AI-assisted task-based language learning influences English proficiency among university students in China. It investigates the mediating roles of cognitive engagement and self-regulated learning, and the moderating role of AI trust. By conceptualizing cognitive engagement and self-regulated learning as complementary cognitive and behavioral pathways, this study provides a mechanism-based explanation of how AI-assisted learning influences language outcomes (Wang et al., 2026; Wang and Reynolds, 2024; Zhang Q. et al., 2025; Zhang Z. et al., 2025). Rather than merely examining whether AI-assisted learning improves language outcomes, the study specifically explains how distinct psychological mechanisms operate within AI-supported task-based learning environments and clarifies the differential explanatory roles of cognitive engagement and self-regulated learning in shaping English proficiency. This integrated framework offers new insights into how AI-enabled learning environments shape language proficiency and extends understanding beyond simple technology–outcome relationships.

### Literature review and hypotheses development

The increasing integration of artificial intelligence into educational environments has reshaped contemporary language learning practices. Universities are increasingly adopting AI-supported learning systems such as intelligent tutoring platforms, automated feedback tools, and adaptive learning environments to enhance students’ learning experiences (Simatupang and Heryono, 2026). Within language education, such technological developments have expanded opportunities for interactive and practice-oriented learning activities that support communicative competence. AI-assisted task-based language learning refers to instructional environments in which artificial intelligence technologies are integrated into task-oriented language activities to provide adaptive feedback, interactive support, and individualized learning experiences during communicative language tasks (Feng, 2025; Huang et al., 2024). Task-based language teaching has long been recognized as an effective pedagogical approach because it engages learners in meaningful language use through purposeful activities. Poli and Morgana (2025) explains that task-based learning encourages learners to apply linguistic knowledge while solving communicative problems, thereby facilitating language acquisition. Extending this pedagogical logic, digital learning environments provide opportunities for individualized practice and immediate feedback, both of which can strengthen learning processes. Feng (2025) demonstrates that technology-enhanced language learning environments enable learners to interact more actively with language tasks and develop stronger learning autonomy. Similarly, Huang et al. (2024) show that digital learning systems can enhance language development by facilitating interaction, feedback, and repeated practice opportunities.

In the context of AI-assisted task-based learning, these features become more pronounced, as learners engage with adaptive feedback and interactive systems that require continuous response, revision, and evaluation of language use (Feng, 2025; Huang et al., 2024). Moreover, such environments shift the learning process from passive content consumption to active task engagement, thereby increasing learners’ involvement in meaning-making and communicative practice. Despite these advancements, empirical understanding of the psychological mechanisms through which AI-assisted task-based learning environments translate into improved English proficiency remains limited. From a theoretical perspective, the present study is primarily grounded in educational psychology and self-regulated learning perspectives, which suggest that instructional environments influence learning outcomes through learners’ internal cognitive and behavioral processes (Zimmerman, 2002; Pintrich, 2000). In AI-assisted learning contexts, learners are not passive recipients of technological support; rather, they actively process AI-generated feedback, regulate learning strategies, and adapt their behaviors during task completion. Therefore, cognitive engagement and self-regulated learning provide important theoretical mechanisms explaining how AI-assisted instructional environments contribute to language learning outcomes.

Within educational psychology, learners’ cognitive engagement and self-regulated learning represent two central processes that explain how instructional environments influence learning outcomes. Cognitive engagement refers to learners’ psychological investment, strategic effort, and active involvement in understanding and processing learning tasks (Fredricks et al., 2004; Reeve, 2013). Hong and Guo (2025) conceptualize cognitive engagement as learners’ investment of effort, strategic thinking, and persistence during learning activities. In language learning contexts, such engagement allows learners to analyze linguistic structures, reflect on communicative tasks, and apply language knowledge more effectively. Ghaffar and Joshua (2025) further show that learning environments encouraging analytical thinking and active participation tend to strengthen students’ cognitive engagement. Recent studies in technology-enhanced language learning have increasingly examined cognitive engagement as a critical mechanism explaining how digital instructional environments influence students’ language learning experiences and academic outcomes (Feng, 2025; Huang et al., 2024). At the same time, self-regulated learning reflects learners’ ability to plan, monitor, and control their learning processes. Zhang Q. et al. (2025) and Zhang Z. et al. (2025) explain that effective learners actively regulate their cognitive and motivational processes while pursuing learning goals. Similarly, Lin (2021) argues that learners who set goals, monitor progress, and adjust strategies demonstrate stronger academic outcomes. When digital technologies support learning activities, students can access flexible learning resources, receive immediate feedback, and monitor their learning progress more effectively. Podosynnikova and Homolia (2025) show that technology-supported learning environments often strengthen learners’ ability to regulate their learning strategies.

Importantly, these two processes represent complementary yet distinct mechanisms: cognitive engagement captures the depth of learners’ information processing, whereas self-regulated learning reflects their ability to manage and adapt learning behaviors over time (Zhang Q. et al., 2025; Zhang Z. et al., 2025; Lin, 2021). In AI-assisted environments, both processes are likely to be activated simultaneously, as learners must not only engage cognitively with AI-generated content but also regulate their interactions with AI tools to optimize learning outcomes. Integrating AI tools within task-based instructional environments may therefore stimulate deeper engagement with learning tasks while simultaneously encouraging learners to monitor and regulate their learning processes. This dual-process perspective provides a stronger theoretical basis for examining how AI-assisted task-based learning influences learner outcomes, rather than treating engagement and self-regulation as isolated effects. Based on this reasoning, the following hypotheses are proposed.

*H1a*: AI-assisted task-based language learning positively influences cognitive engagement.

*H1b*: AI-assisted task-based language learning positively influences self-regulated learning.

Zhou and Hou (2024) demonstrate that students who invest greater cognitive effort in learning tasks tend to achieve stronger academic outcomes. Likewise, Feng (2025) shows that cognitively engaged learners apply deeper analytical thinking and problem-solving strategies, which contribute to improved learning performance. In language learning contexts, such engagement enables learners to process linguistic input more effectively and apply language knowledge during communicative tasks (Roozafzai, 2024). This suggests that cognitive engagement enhances proficiency not merely through effort, but through deeper processing, integration, and application of linguistic knowledge, which are essential for developing communicative competence (Feng, 2025; Huang et al., 2024).

In addition to engagement, self-regulated learning has been widely recognized as a critical predictor of academic success. For instance, Shi et al. (2025) illustrates that students who monitor their learning progress and regulate their learning strategies demonstrate stronger academic achievement. Zhang Q. et al. (2025) and Zhang Z. et al. (2025) further explains that self-regulated learners are more capable of sustaining motivation and adapting learning strategies in language learning contexts, which contributes to improved language proficiency. Unlike cognitive engagement, which reflects depth of processing during tasks, self-regulated learning reflects learners’ ability to sustain, organize, and adapt their learning over time, thereby ensuring consistent progress and effective use of learning opportunities (Zhang Q. et al., 2025; Zhang Z. et al., 2025; Lin, 2021). Accordingly, the following hypotheses are proposed.

*H2a*: Cognitive engagement positively influences English proficiency.

*H2b*: Self-regulated learning positively influences English proficiency.

In technology-enhanced learning environments, engagement and self-regulated learning frequently function as mediating mechanisms that translate instructional experiences into improved academic outcomes. Zhou and Hou (2024) demonstrate that instructional environments encouraging active participation stimulate engagement, which subsequently enhances learning performance. Similarly, Shi et al. (2025), Zhang Q. et al. (2025), and Zhang Z. et al. (2025) validate that digital learning environments often improve academic outcomes by strengthening learners’ self-regulatory capabilities. When AI technologies are integrated into task-based instructional environments, learners receive adaptive feedback, structured task guidance, and opportunities for individualized learning pathways. Such features may encourage deeper task engagement and support the regulation of learning behaviors, both of which can contribute to improved language proficiency.

More importantly, AI-assisted environments may intensify this indirect process, as learners are continuously exposed to feedback, suggestions, and alternative language outputs, which require both active cognitive engagement and ongoing self-regulation to be effectively utilized (Feng, 2025; Zhang Q. et al., 2025; Zhang Z. et al., 2025). In this process, cognitive engagement enables learners to actively analyze, interpret, and apply AI-generated linguistic feedback during communicative tasks, thereby improving the depth of language processing and comprehension (Huang et al., 2024). At the same time, self-regulated learning allows learners to continuously monitor their progress, adjust learning strategies, and utilize AI-supported guidance more effectively throughout the learning process (Zhang Q. et al., 2025; Zhang Z. et al., 2025; Lin, 2021). Therefore, AI-assisted task-based learning is expected to influence English proficiency indirectly through these underlying cognitive and behavioral mechanisms rather than through direct instructional exposure alone (Shi et al., 2025; Zhou and Hou, 2024). This implies that the influence of AI-assisted task-based learning on proficiency is unlikely to be direct, but instead operates through these underlying psychological mechanisms. Building on these insights, the following mediation hypotheses are proposed.

*H3a*: Cognitive engagement mediates the relationship between AI-assisted task-based language learning and English proficiency.

*H3b*: Self-regulated learning mediates the relationship between AI-assisted task-based language learning and English proficiency.

While instructional design and learner processes play central roles in shaping learning outcomes, the effectiveness of AI-supported learning environments may also depend on learners’ perceptions of the technology itself. According to Akman Yeşilel (2025), trust represents a critical factor influencing individuals’ willingness to rely on technological systems. Besides, Nazaretsky et al. (2022) demonstrate that trust significantly influences users’ acceptance and use of technological systems, particularly when automated recommendations are involved. In addition, Wu et al. (2022) explain that trust determines the extent to which individuals effectively collaborate with artificial intelligence systems. Within educational contexts, learners who perceive AI tools as reliable are more likely to rely on AI-generated feedback and incorporate it into their learning processes. Similarly, Alyoussef et al. (2025) show that trust functions as a key determinant of technology adoption and utilization. Further, Ma et al. (2023) highlights that trust is essential for fostering positive human–AI interaction and ensuring users’ willingness to depend on AI-supported decision systems. Recent work by Zhang Q. et al. (2025) and Zhang Z. et al. (2025) also emphasizes that users’ trust in AI systems significantly shapes their engagement and acceptance of AI-driven technologies. Consequently, learners who exhibit higher levels of trust in AI systems may benefit more from AI-assisted learning environments.

Importantly, trust is more likely to influence how learners translate AI-assisted learning experiences into performance outcomes, rather than whether they initially engage with or regulate their learning processes. That is, while cognitive engagement and self-regulated learning are primarily activated by task design and instructional features (Feng, 2025; Zhang Q. et al., 2025; Zhang Z. et al., 2025), trust determines the extent to which learners rely on, internalize, and effectively utilize AI-generated feedback in producing learning outcomes (Nazaretsky et al., 2022; Ma et al., 2023). In this sense, trust operates as a boundary condition at the outcome stage, strengthening or weakening the impact of AI-assisted learning on English proficiency.

Therefore, AI trust may strengthen the relationship between AI-assisted task-based language learning and English proficiency. Accordingly, the following hypothesis is proposed.

**Figure 1:**
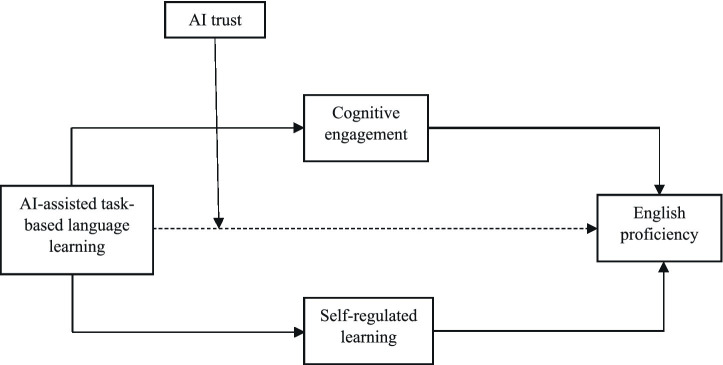
Conceptual model.

*H4*: AI trust positively moderates the relationship between AI-assisted task-based language learning and English proficiency, such that the relationship is stronger when AI trust is high (shown in Figure 1).

### Methodology

The study adopted a quantitative cross-sectional research design to investigate the relationships among AI-assisted task-based language learning, cognitive engagement, self-regulated learning, English proficiency, and AI trust among university students in China. The Chinese higher education context was considered particularly appropriate because English proficiency plays an increasingly important role in students’ academic achievement, international academic engagement, and future employability within globally competitive educational and professional environments (Li et al., 2024; Zhang Q. et al., 2025; Zhang Z. et al., 2025). In addition, Chinese universities have increasingly integrated AI-supported technologies into English language instruction, making this context suitable for examining AI-assisted learning processes. The research context consisted of public universities where English is taught as a compulsory foreign language course and where instructors increasingly integrate digital tools and AI-supported resources into task-based learning activities. In this study, AI-assisted task-based language learning refers to students’ perceived exposure to and interaction with AI-supported instructional activities within classroom-based task environments, rather than an objective measure of instructional implementation. This distinction is important, as the study focuses on learners’ psychological responses to AI-supported learning experiences. A survey-based approach was considered appropriate because it allows the systematic collection of perceptual and behavioral data from a large number of respondents within a natural educational setting, which is consistent with established practices in educational and behavioral research (Creswell and Creswell, 2018).

Participants were selected using a non-probability convenience sampling technique. This sampling approach is commonly applied in educational studies where access to specific student populations is facilitated through institutional cooperation and classroom-based data collection (Etikan et al., 2016). Data were collected from undergraduate students enrolled in English-related courses at four public universities located in eastern and central China. Students enrolled in English-related courses were considered especially relevant for the present study because they regularly engage in language-learning activities requiring communicative practice, feedback utilization, and continuous interaction with digital language-learning resources, thereby providing an appropriate context for examining AI-assisted task-based language learning and English proficiency development. These institutions had incorporated AI-assisted learning activities within task-based English instruction as part of their regular coursework. Specifically, students interacted with AI-supported tools such as automated grammar and feedback systems, AI-assisted writing platforms, AI-supported speaking and pronunciation applications, and generative AI tools including ChatGPT for language practice and task completion. These AI tools were integrated into classroom activities including collaborative writing exercises, vocabulary tasks, reading comprehension activities, speaking simulations, and feedback-oriented revision exercises. Students typically engaged with these AI-supported systems on a weekly basis during scheduled English learning sessions, where the tools were used to provide immediate feedback, language correction, task guidance, and individualized learning support. A total of 500 paper-based questionnaires were distributed with the assistance of course instructors during scheduled class sessions. Students were approached at the end of their lectures and invited to voluntarily participate in the study. Each questionnaire packet included a brief cover letter explaining the purpose of the research, assuring respondents that participation was voluntary, and emphasizing that their responses would remain anonymous and confidential. The cover letter also clarified that the collected information would be used strictly for academic research purposes and that respondents could withdraw from the study at any time.

Out of the 500 distributed questionnaires, 452 were returned. After screening the responses for missing values, incomplete answers, and patterned responses, 412 usable questionnaires remained for final analysis. This resulted in an effective response rate of approximately 82.4 percent. The sample size satisfies recommended thresholds for structural equation modeling, which suggest that samples exceeding 300 observations provide sufficient statistical power and stable parameter estimates for complex mediation and moderation models (Hair et al., 2022). Furthermore, although the cross-sectional design enables efficient data collection, it may introduce concerns related to common method bias due to the use of self-reported measures from a single source. To address this issue, statistical procedures were applied in subsequent analyses to assess the potential impact of common method bias.

The demographic characteristics of the respondents indicate a diverse student representation. Approximately 53 percent of participants were female and 47 percent were male. In terms of academic year, 27 percent were first-year students, 33 percent were second-year students, 25 percent were third-year students, and 15 percent were fourth-year students. Regarding age distribution, 35 percent of respondents were between 18 and 19 years old, 43 percent were between 20 and 21 years old, and the remaining 22 percent were aged 22 years or above. With respect to English learning experience, 46 percent of students reported studying English for more than 10 years, while 54 percent indicated between 6 and 10 years of prior English learning experience. These characteristics suggest that respondents possess sufficient exposure to formal English learning contexts, making them appropriate for examining the effects of AI-assisted task-based language learning on language proficiency. These demographic characteristics suggest that the sample provides an appropriate representation of university students actively engaged in English language learning in China.

### Measures

All constructs were measured using previously validated scales and were slightly adapted to fit the context of AI-assisted task-based English learning. Responses were recorded on a five-point Likert scale ranging from 1 (strongly disagree) to 5 (strongly agree). Minor wording modifications were introduced to ensure contextual relevance to AI-supported language learning environments, such as incorporating references to AI tools, task-based activities, and English learning contexts while preserving the original meaning of scale items (see Appendix A).

AI-assisted task-based language learning was assessed using eight items adapted from task-based language learning and technology-enhanced learning scales (Ellis, 2003; Lai and Zheng, 2018). A sample item is “AI-supported tasks help me practice English in realistic communication situations.” The items were reworded to reflect students’ perceived interaction with AI-supported tasks rather than general instructional practices.

Cognitive engagement was measured with five items adapted from the student engagement literature (Fredricks et al., 2004; Reeve, 2013). A sample item is “I try to understand the underlying meaning of English tasks rather than just completing them.” The scale captures learners’ depth of processing, effort investment, and strategic thinking during AI-assisted language tasks.

Self-regulated learning was measured with five items adapted from established self-regulated learning scales (Pintrich, 2000; Zimmerman, 2002). A sample item is “I set goals for improving my English when completing learning tasks.” Items were contextualized to reflect learners’ planning, monitoring, and regulation behaviors within AI-supported learning environments.

English proficiency was measured using five items adapted from perceived language proficiency scales used in second language research (Dewaele and MacIntyre, 2014). A sample item is “I can effectively express my ideas in English during academic activities.” The measure reflects students’ perceived communicative competence and ability to use English effectively in academic contexts.

AI trust was measured using five items adapted from prior research on trust in artificial intelligence systems (McKnight et al., 2011). A sample item is “I trust the suggestions provided by AI tools when learning English.” The items capture learners’ confidence in the reliability, usefulness, and credibility of AI-supported learning systems.

### Control

To reduce the potential influence of alternative explanations, demographic variables including gender, age, academic year, and prior English learning experience were included as control variables in the analysis. These factors were controlled because previous studies have suggested that learners’ demographic and educational backgrounds may influence language learning behaviors, technology usage patterns, and English proficiency outcomes in higher education contexts.

### Results

The study employed partial least squares structural equation modeling (PLS-SEM) for data analysis. This approach was considered appropriate given the study’s objective of examining complex relationships involving multiple mediating and moderating effects, as well as its focus on maximizing the explained variance of endogenous constructs. PLS-SEM is particularly suitable for prediction-oriented research and for models that emphasize the identification of key driver relationships rather than strict theory confirmation (Hair et al., 2022). Furthermore, the model includes parallel mediation and moderation effects, which increases model complexity and supports the use of PLS-SEM as a robust analytical technique. Therefore, PLS-SEM was deemed appropriate for estimating the proposed model and assessing both measurement and structural relationships.

First of all, the study assesses the means, standard deviations, and Pearson correlation coefficients among the study constructs (Appendix B). The results show that all constructs are positively and significantly correlated with one another, providing preliminary support for the proposed theoretical relationships. The mean values indicate generally favorable perceptions of AI-assisted task-based language learning, AI trust, cognitive engagement, self-regulated learning, and English proficiency, while the standard deviations suggest adequate variability in respondents’ responses.

Subsequently, the study analyzes the measurement properties of the constructs by examining indicator loadings, internal consistency reliability, and convergent validity (shown in Table 1). The results indicate that most item loadings exceed the commonly recommended threshold of 0.60, demonstrating acceptable indicator reliability for the constructs. Although a small number of items exhibited relatively lower loadings, they were retained due to their theoretical relevance and contribution to content validity, which is consistent with recommended practices in PLS-SEM when overall construct reliability is satisfactory (Hair et al., 2022). In particular, following the recommendation of Hair et al. (2022), a sensitivity analysis was conducted for AIL1 due to its loading below 0.70. The temporary removal of the item resulted in only marginal improvements in composite reliability and average variance extracted values, while the overall structural relationships and substantive interpretation of the model remained unchanged. Therefore, the item was retained to preserve the conceptual comprehensiveness and content validity of the construct. Internal consistency reliability was evaluated using Cronbach’s alpha and composite reliability values. The findings show that Cronbach’s alpha values range from 0.747 to 0.852, while composite reliability values range from 0.823 to 0.887, both exceeding the recommended minimum threshold of 0.70, thereby confirming satisfactory internal consistency.

**Table 1:** Descriptive stats and correlations.

Variables	Mean	SD	1	2	3	4	5
1. AI-Assisted task-based language learning	3.84	0.71	—				
2. Cognitive engagement	3.76	0.68	0.42**	—			
3. Self-regulated learning	3.89	0.65	0.50**	0.47**	—		
4. English proficiency	3.73	0.74	0.56**	0.39**	0.61**	—	
5. AI trust	3.81	0.69	0.48**	0.36**	0.44**	0.53**	—

Convergent validity was assessed through the average variance extracted (AVE), with all constructs reaching the minimum acceptable value of 0.50, indicating that the constructs explain at least half of the variance of their indicators. Although some AVE values are close to the threshold, they remain within acceptable limits, suggesting adequate convergence without compromising measurement quality. These results suggest that the measurement model demonstrates adequate reliability and convergent validity, supporting the suitability of the constructs for further structural analysis in line with recommended PLS-SEM evaluation criteria (Hair et al., 2022).

In addition, the study examines the potential issue of multicollinearity among the constructs by analyzing variance inflation factor (VIF) values (shown in Table 2). Multicollinearity can bias path estimates and inflate standard errors, making it important to ensure that predictor variables are not excessively correlated. The results reveal that the VIF values range from 1.000 to 2.791, which remain well below the commonly accepted threshold of 3.3 suggested for PLS-SEM models. These values indicate that multicollinearity is not a concern in the proposed model and that the predictor constructs are sufficiently distinct from one another (Hair et al., 2022). Furthermore, these VIF values also provide additional evidence against potential common method bias, as values below 3.3 suggest that collinearity arising from a single-source measurement is unlikely to distort the results.

**Table 2:** Psychometric properties.

Constructs (Items)	Loadings	CA	CR	AVE
*AI-assisted task-based language learning*		0.852	0.887	0.500
AIL1	0.509			
AIL2	0.685			
AIL3	0.696			
AIL4	0.767			
AIL5	0.795			
AIL6	0.753			
AIL7	0.753			
AIL8	0.645			
*AI trust*		0.821	0.874	0.582
AIT1	0.712			
AIT2	0.831			
AIT3	0.746			
AIT4	0.752			
AIT5	0.767			
*Cognitive engagement*		0.774	0.845	0.522
CE1	0.711			
CE2	0.693			
CE3	0.673			
CE4	0.728			
CE5	0.800			
*English proficiency*		0.816	0.873	0.580
EP1	0.694			
EP2	0.828			
EP3	0.781			
EP4	0.824			
EP5	0.666			
*Self-regulated learning*		0.747	0.823	0.500
SL1	0.676			
SL2	0.744			
SL3	0.582			
SL4	0.763			
SL5	0.699			

To further assess the potential impact of common method bias, Harman’s single-factor test was conducted. The results revealed that the first factor accounted for 34.27% of the total variance, which remains below the commonly recommended threshold of 50%, suggesting that common method bias is unlikely to pose a serious concern in this study. Although Harman’s single-factor test has recognized limitations as a standalone diagnostic procedure, it remains a widely used preliminary assessment in behavioral and educational research. Furthermore, together with the VIF values below the recommended threshold, the findings provide additional confidence that common method bias is unlikely to substantially distort the observed relationships.

Furthermore, the study assesses discriminant validity using the heterotrait–monotrait (HTMT) ratio, which is considered one of the most reliable criteria for evaluating construct distinctiveness in variance-based structural equation modeling (shown in Table 3). The HTMT values among all constructs range from 0.363 to 0.857, remaining below the recommended threshold of 0.90. This indicates that each construct is empirically distinct from the others and captures a unique conceptual domain within the research model. Establishing discriminant validity is crucial because it confirms that the constructs measure different phenomena rather than overlapping dimensions. The findings therefore demonstrate that the model satisfies the HTMT criterion, confirming adequate discriminant validity and supporting the robustness of the measurement model (Henseler et al., 2015; Hair et al., 2022).

**Table 3:** Variance inflation factor (VIF).

Constructs	AIL	AIT	CE	EP	SL
AIL			1.000	2.171	1.000
AIT				2.791	
CE				1.205	
EP					
SL				1.543	

**Figure 2:**
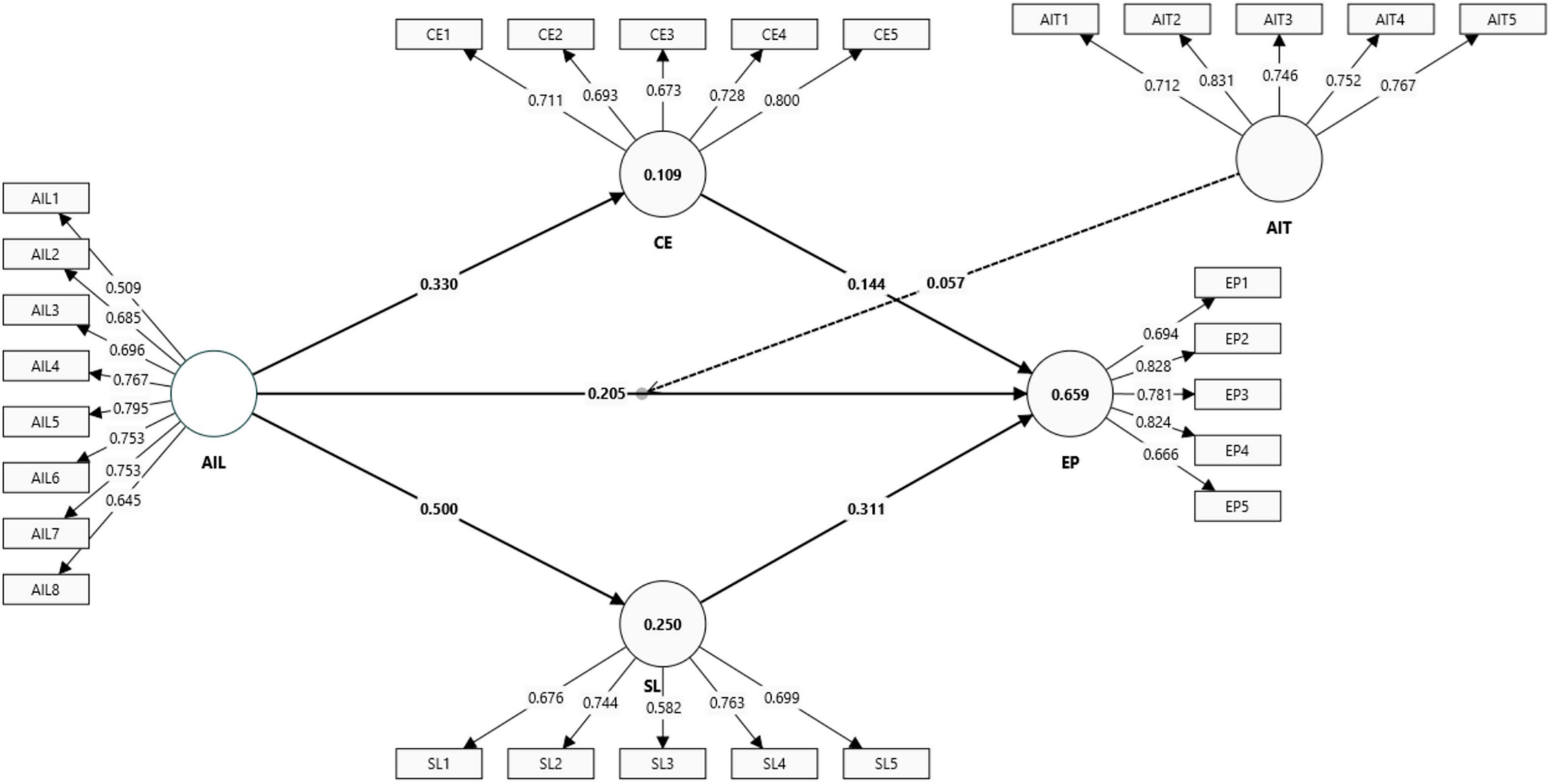
Structural equation model.

The study subsequently evaluates the structural model by examining the path coefficients, t-values, and significance levels to test the proposed hypotheses (shown in Table 4 and Figure 2). The findings indicate that AI-assisted task-based language learning has a positive and significant effect on cognitive engagement (*β* = 0.330, *t* = 4.856, *p* < 0.001), suggesting that AI-supported learning tasks enhance students’ cognitive involvement in English learning activities. Therefore, Hypothesis 1a is supported. In addition, AI-assisted task-based language learning shows a strong and significant influence on self-regulated learning (*β* = 0.500, *t* = 12.887, *p* < 0.001), indicating that AI-supported tasks encourage learners to plan, monitor, and regulate their learning behaviors. Notably, the stronger effect on self-regulated learning compared to cognitive engagement suggests that AI-assisted environments may be particularly effective in structuring and guiding learners’ learning processes rather than solely enhancing deep cognitive processing. Thus, Hypothesis 1b is also supported.

**Table 4:** Discriminant validity (HTMT ratio).

Constructs	AIL	AIT	CE	EP	SL
AIL					
AIT	0.813				
CE	0.385	0.443			
EP	0.786	0.857	0.548		
SL	0.548	0.598	0.363	0.762	

Furthermore, the results demonstrate that cognitive engagement positively affects English proficiency (*β* = 0.144, *t* = 3.270, *p* = 0.001), implying that deeper intellectual involvement in learning activities contributes to improved language performance. However, the relatively modest effect size indicates that while cognitive engagement is important, its direct contribution to proficiency may be limited compared to other learning mechanisms. Hence, Hypothesis 2a is supported. Similarly, self-regulated learning shows a significant positive effect on English proficiency (*β* = 0.311, *t* = 8.446, *p* < 0.001), indicating that students who actively regulate their learning strategies tend to achieve higher language proficiency. The stronger effect of self-regulated learning further highlights its critical role in sustaining learning outcomes over time, particularly in technology-supported environments. Therefore, Hypothesis 2b is supported.

The study also assesses the mediating effects. The indirect effect of AI-assisted task-based language learning on English proficiency through cognitive engagement is significant (*β* = 0.047, *t* = 2.314, *p* = 0.021), confirming that cognitive engagement partially mediates this relationship, thereby supporting Hypothesis 3a. Likewise, self-regulated learning significantly mediates the relationship between AI-assisted task-based language learning and English proficiency (*β* = 0.156, *t* = 8.247, *p* < 0.001), indicating that improved self-regulation explains part of the impact of AI-assisted learning on language outcomes. Importantly, the substantially stronger indirect effect through self-regulated learning compared to cognitive engagement suggests that AI-assisted learning primarily influences language proficiency through behavioral regulation mechanisms rather than purely cognitive processing pathways. Thus, Hypothesis 3b is supported (see Table 4).

**Figure 3:**
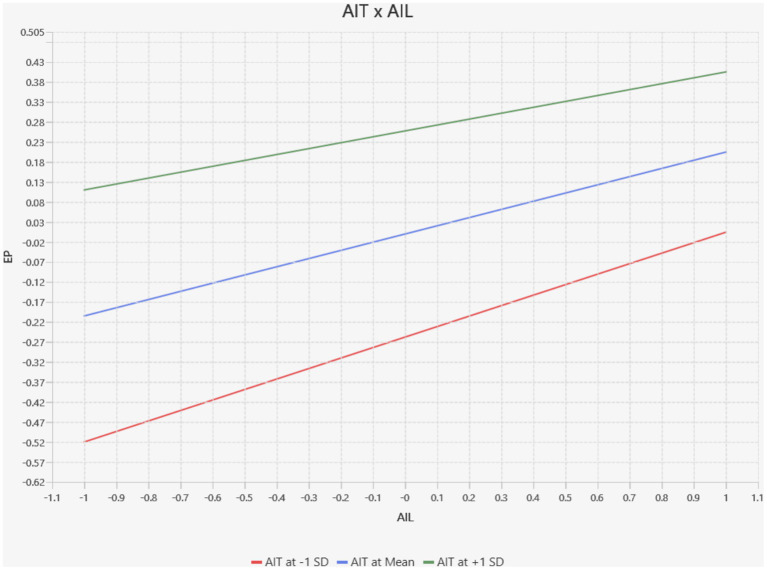
Simple slopes of the AIT × AIL interaction on English proficiency.

Finally, the moderating effect of AI trust on the relationship between AI-assisted task-based language learning and English proficiency was examined (interaction effect shown in Figure 3). The results reveal that the interaction term is positive and statistically significant (*β* = 0.057, *t* = 2.225, *p* = 0.027), indicating that AI trust strengthens the positive influence of AI-assisted task-based language learning on English proficiency. Although the magnitude of the moderating effect is relatively small, its significance suggests that trust functions as a subtle but meaningful boundary condition that enhances the effectiveness of AI-supported learning environments. Accordingly, Hypothesis 4 is supported. The graphical representation of the moderation effect further clarifies this relationship. As illustrated in Figure 3, the slope representing the relationship between AI-assisted task-based language learning and English proficiency becomes steeper at higher levels of AI trust, whereas the relationship appears comparatively weaker when AI trust is low. This pattern suggests that students who have greater confidence in AI tools are more likely to benefit from AI-supported task-based learning activities in terms of improving their English proficiency.

It should also be noted that the negative intercept observed for the low AI trust condition (−1 SD) in Figure 3 reflects the standardized nature of the plotted values rather than the presence of negative English proficiency outcomes in absolute terms. Specifically, the simple-slopes graph was generated using standardized latent variable scores, where values below zero indicate below-average levels relative to the sample mean. The pattern nevertheless suggests that learners with comparatively lower trust in AI systems and lower exposure to AI-assisted task-based learning benefit less from AI-supported instructional environments in terms of English proficiency development.

In addition to statistical significance, the effect size (f^2^) values were examined to assess the substantive contribution of each predictor construct. The results indicate that AI-assisted task-based language learning demonstrates a moderate-to-substantial effect on self-regulated learning (f^2^ = 0.334) and a small-to-moderate effect on cognitive engagement (f^2^ = 0.122). Furthermore, self-regulated learning exhibits a moderate effect on English proficiency (f^2^ = 0.184), whereas cognitive engagement shows a relatively smaller effect (f^2^ = 0.050). The moderating effect of AI trust on the relationship between AI-assisted task-based language learning and English proficiency is small but meaningful (f^2^ = 0.020), which is consistent with prior recommendations for interpreting moderation effects in behavioral research.

Finally, the study evaluates the predictive relevance of the model using the PLS-Predict procedure. The results indicate that all endogenous constructs demonstrate positive Q^2^predict values, suggesting that the model possesses adequate predictive capability. Specifically, English proficiency shows the highest predictive relevance (Q^2^predict = 0.540), followed by self-regulated learning (Q^2^predict = 0.241) and cognitive engagement (Q^2^predict = 0.091). The relatively lower predictive relevance for cognitive engagement suggests that additional factors beyond AI-assisted learning may influence this construct, indicating that cognitive engagement is shaped by a broader set of learning conditions. The corresponding RMSE and MAE values further indicate acceptable prediction errors across the constructs. Overall, these findings suggest that the proposed model has satisfactory out-of-sample predictive power and is capable of effectively predicting the key endogenous variables in the study (Hair et al., 2022) (see Tables 5, 6).

**Table 5:** Path coefficients.

Relationships	Original sample (O)	Standard deviation (STDEV)	T statistics (|O/STDEV|)	*p* values	f^2^	Decision
AIL→CE (*H1a*)	0.330	0.068	4.856	0.000	0.122	Supported
AIL→SL (*H1b*)	0.500	0.039	12.887	0.000	0.334	Supported
CE→EP (*H2a*)	0.144	0.044	3.270	0.001	0.050	Supported
SL→EP (*H2b*)	0.311	0.037	8.446	0.000	0.184	Supported
AIL→CE→EP (*H3a*)	0.047	0.020	2.314	0.021		Supported
AIL→SL→EP (*H3b*)	0.156	0.019	8.247	0.000		Supported
AIT × AIL→EP (*H4*)	0.057	0.026	2.225	0.027	0.020	Supported

**Table 6:** PLS Predict.

Constructs	Q^2^predict	RMSE	MAE
CE	0.091	0.962	0.747
EP	0.540	0.686	0.491
SL	0.241	0.876	0.728

Last but not the least, the explanatory power of the model was assessed using *R*^2^ values. The results indicate that AI-assisted task-based language learning explains 10.9% of the variance in cognitive engagement (*R*^2^ = 0.109) and 25.0% of the variance in self-regulated learning (*R*^2^ = 0.250). Furthermore, the model explains 65.9% of the variance in English proficiency (*R*^2^ = 0.659), indicating strong explanatory power for the primary outcome construct. Notably, the comparatively lower R^2^ value for cognitive engagement suggests that AI-assisted task-based learning exerts a relatively limited influence on learners’ deep cognitive processing compared to its stronger influence on behavioral regulation processes. This finding is consistent with the broader pattern of results observed in the study, where self-regulated learning demonstrated substantially stronger direct and mediating effects than cognitive engagement. Therefore, the findings suggest that AI-assisted learning environments may primarily operate through facilitating learners’ planning, monitoring, and adaptive learning behaviors rather than substantially transforming deeper cognitive engagement processes. Overall, the R^2^ values suggest that the proposed model provides acceptable explanatory power for the key endogenous constructs.

## Discussion

The primary objective of this study was to examine how AI-assisted task-based language learning influences English proficiency among university students and to explore the underlying psychological mechanisms that explain this relationship. Specifically, the study aimed to investigate whether cognitive engagement and self-regulated learning mediate the relationship between AI-assisted task-based learning and English proficiency, and whether AI trust strengthens this relationship. The findings demonstrate that the proposed objectives were successfully achieved, as all hypothesized relationships were statistically supported. More importantly, the results provide a clearer understanding of how AI-assisted learning environments operate through distinct cognitive and behavioral mechanisms rather than producing direct effects on learning outcomes.

First, the results reveal that AI-assisted task-based language learning significantly enhances both cognitive engagement and self-regulated learning among students. This finding aligns with prior studies emphasizing that technology-supported learning environments can stimulate deeper cognitive involvement and active participation in learning tasks (Kumar et al., 2024; Ng et al., 2024). Previous research has shown that task-based instructional approaches encourage learners to actively process information and apply language knowledge in meaningful contexts (Shi et al., 2025), which promotes engagement and reflective learning behaviors. Similarly, studies on digital learning environments have reported that AI-supported instructional tools can encourage learners to adopt more autonomous and self-directed learning strategies (Li et al., 2024; Younas et al., 2025). However, the findings extend prior research by showing that AI-assisted environments do not equally influence all learning processes, as the effect on self-regulated learning is substantially stronger than on cognitive engagement. This suggests that AI-supported task environments may be particularly effective in structuring learners’ learning behaviors—such as planning, monitoring, and strategy adjustment—rather than solely enhancing deep cognitive processing.

Second, the findings indicate that both cognitive engagement and self-regulated learning significantly contribute to improving English proficiency. This result is consistent with earlier research in language education and educational psychology (Wu et al., 2022), which highlights that students who invest greater cognitive effort in learning tasks and actively regulate their learning strategies tend to achieve stronger academic outcomes. Previous studies have also demonstrated that learners who monitor their progress, set learning goals, and adjust their strategies during language learning activities are more likely to develop higher levels of language competence (Navas Bonilla et al., 2025; Wang et al., 2024). Nevertheless, the comparatively stronger effect of self-regulated learning suggests that sustained behavioral control and strategic adaptation play a more critical role in language proficiency than momentary cognitive engagement. This implies that while engagement facilitates deeper understanding, long-term proficiency gains may depend more heavily on learners’ ability to regulate and manage their learning processes over time.

Furthermore, the mediation analysis confirms that cognitive engagement and self-regulated learning serve as important mechanisms linking AI-assisted task-based language learning with English proficiency. This finding supports earlier research suggesting that instructional approaches influence learning outcomes indirectly through students’ cognitive and motivational processes (Feng, 2025; Ghaffar and Joshua, 2025; Zhou and Hou, 2024). Prior studies in technology-enhanced learning have similarly found that digital learning environments improve academic outcomes by fostering deeper engagement and self-regulated learning behaviors (Hong and Guo, 2025; Huang et al., 2024). Importantly, the stronger indirect effect through self-regulated learning indicates that AI-assisted learning primarily operates through behavioral regulation pathways rather than purely cognitive mechanisms. This highlights the role of AI tools as facilitators of structured learning routines, continuous feedback utilization, and adaptive learning strategies.

Finally, the results show that AI trust strengthens the relationship between AI-assisted task-based learning and English proficiency. This finding is consistent with previous research on trust in artificial intelligence (Ma et al., 2023; Nazaretsky et al., 2022), which suggests that learners who perceive AI systems as reliable and useful are more likely to rely on them during learning activities and consequently benefit more from technology-supported instruction. However, the relatively modest magnitude of the moderating effect suggests that trust functions as a subtle boundary condition rather than a dominant driver of learning outcomes. This indicates that while trust enhances the effectiveness of AI-assisted learning, the primary mechanisms of influence remain rooted in engagement and self-regulation processes. These findings, however, extend existing literature by demonstrating that AI-assisted learning environments do not operate through uniform psychological mechanisms. Specifically, the results reveal that self-regulated learning represents a comparatively stronger explanatory pathway than cognitive engagement, suggesting that AI-supported instructional environments may influence English proficiency more effectively through facilitating learners’ behavioral regulation, adaptive learning strategies, and continuous feedback utilization rather than solely enhancing deep cognitive processing (Zhang Q. et al., 2025; Zhang Z. et al., 2025; Lin, 2021). In addition, the study clarifies that AI trust functions as a boundary condition that strengthens the effectiveness of AI-assisted learning environments, thereby providing a more nuanced mechanism-based explanation of how AI-supported educational systems influence language learning outcomes (Nazaretsky et al., 2022; Ma et al., 2023).

### Theoretical implications

The findings of this study offer several theoretical contributions to the literature on technology-enhanced language learning and learner psychology. First, the study expands existing research on task-based language learning by integrating artificial intelligence into the instructional framework, thereby extending traditional pedagogical models toward AI-assisted learning environments. While previous studies have established that task-based learning improves language outcomes through meaningful interaction and active language use (Lume and Hisbullah, 2022; Oliver and Bogachenko, 2024), the present findings demonstrate that the integration of AI tools can further enhance these instructional effects by stimulating learners’ cognitive engagement and self-regulated learning processes. More importantly, the study advances theoretical understanding by demonstrating that AI-assisted instructional environments operate through distinct yet complementary psychological mechanisms, rather than producing direct effects on learning outcomes. In doing so, the study advances theoretical discussions on technology-enhanced learning by identifying the psychological mechanisms through which AI-supported instructional approaches influence language proficiency. Specifically, the results extend prior engagement and self-regulated learning theories by showing that these cognitive processes function as key explanatory pathways linking AI-assisted pedagogical practices with language learning outcomes. This mechanism-based perspective moves beyond traditional instructional models by clarifying how AI-supported environments activate both deep cognitive processing and sustained behavioral regulation.

Importantly, the findings further refine theoretical understanding by demonstrating that these mechanisms do not contribute equally to learning outcomes. The comparatively stronger role of self-regulated learning suggests that AI-assisted learning environments may primarily operate through behavioral adaptation and strategic learning management rather than solely through heightened cognitive involvement. This distinction advances existing engagement and self-regulation theories by indicating that AI-supported instructional systems may strengthen learners’ capacity to organize, monitor, and adapt their learning behaviors more effectively than their deep cognitive processing alone (Zhang Q. et al., 2025; Zhang Z. et al., 2025; Lin, 2021).

Moreover, the study contributes to the emerging literature on artificial intelligence in education by incorporating AI trust as a boundary condition that shapes the effectiveness of AI-supported learning environments. While previous research has often examined AI adoption from technological or system design perspectives (Ghaffar and Joshua, 2025; Kumar et al., 2024), the current findings highlight the importance of learners’ psychological perceptions of AI systems in determining the educational benefits derived from them. By simultaneously integrating instructional design, learner cognitive processes, and technology trust within a single analytical framework, the study broadens theoretical understanding of how AI-enabled learning environments operate and provides a more comprehensive explanation of the mechanisms through which AI-assisted language learning can enhance students’ language proficiency. Importantly, the findings demonstrate that trust does not function as a primary driver of learning outcomes but rather as a boundary condition that strengthens or weakens the translation of AI-assisted learning experiences into actual performance gains. This distinction refines existing theories of technology acceptance by positioning trust within the outcome realization stage rather than the initial adoption stage. Accordingly, the study shifts theoretical attention from viewing AI trust merely as an antecedent of technology adoption toward understanding trust as a contextual mechanism influencing the effectiveness of AI-supported learning processes and outcome realization (Nazaretsky et al., 2022; Ma et al., 2023).

Rather than emphasizing the novelty of combining variables, the present study contributes by offering a theoretically integrated explanation of how AI-assisted task-based learning influences language proficiency through interconnected cognitive, behavioral, and perceptual mechanisms. By distinguishing the relative roles of cognitive engagement, self-regulated learning, and AI trust, the study provides a more nuanced understanding of AI-enabled learning processes and clarifies the conditions under which such instructional approaches are most effective. Thus, the study contributes not only by identifying significant relationships, but also by refining theoretical understanding of how AI-assisted educational environments operate through differentiated psychological pathways and conditional trust-based mechanisms.

### Practical implications

The findings of this study provide several practical implications for educators, institutions, and developers of AI-supported learning technologies. First, language instructors should actively integrate AI-assisted task-based activities into English language courses, as the results indicate that such approaches enhance both cognitive engagement and self-regulated learning. Designing classroom tasks that incorporate AI tools for interactive practice, feedback, and problem-solving can help students engage more deeply with language learning activities. Importantly, given that the findings show a stronger effect on self-regulated learning than cognitive engagement, instructors should particularly emphasize structured learning activities that guide students’ planning, monitoring, and strategy use during AI-assisted tasks.

Second, educators should intentionally design learning environments that promote cognitive engagement during AI-supported tasks. For example, instructors can encourage students to analyze, reflect, and apply language concepts during AI-assisted exercises rather than focusing solely on task completion. Such strategies can strengthen the cognitive processes that contribute to improved language proficiency. This is especially important because the results suggest that cognitive engagement plays a meaningful but comparatively smaller role, indicating that deeper processing needs to be deliberately stimulated rather than assumed to occur automatically in AI-supported environments.

Third, universities and language programs should incorporate training sessions that help students develop self-regulated learning strategies when using AI learning tools. Guiding students on how to set learning goals, monitor their progress, and adjust learning strategies while interacting with AI systems can maximize the educational benefits of AI-assisted learning environments. Given the strong influence of self-regulated learning on English proficiency, such interventions can significantly enhance students’ long-term learning outcomes and ensure sustained improvement beyond individual tasks.

Fourth, institutions should focus on building students’ trust in AI learning tools by ensuring transparency, reliability, and clear guidance on how AI systems generate feedback or recommendations. When students perceive AI tools as credible and useful, they are more likely to rely on them effectively during learning tasks. However, considering that the moderating effect of AI trust is relatively modest, institutions should treat trust as a supporting condition rather than a primary driver, ensuring that instructional design and learner processes remain the central focus of AI-assisted learning strategies.

Finally, developers of educational AI technologies should design user-friendly systems that provide adaptive feedback, personalized task recommendations, and supportive learning guidance. Such features can enhance both learner engagement and self-regulated learning behaviors, thereby improving the effectiveness of AI-supported language learning environments. In particular, AI systems should be designed to not only deliver feedback but also actively support learners’ regulation processes, such as prompting goal setting, progress monitoring, and adaptive strategy use.

### Limitations and directions for future studies

Several limitations should be acknowledged when interpreting the findings of this study. First, the study employed a cross-sectional research design, which limits the ability to draw causal inferences among the examined variables. Although the results provide strong evidence of associations, the temporal ordering of AI-assisted learning, cognitive engagement, self-regulated learning, and English proficiency cannot be conclusively established. Future research may adopt longitudinal or experimental designs to better capture the dynamic relationships between AI-assisted learning environments and language proficiency over time.

Second, the data were collected from university students in China, which may restrict the generalizability of the findings to other educational contexts or cultural settings. Given that learning behaviors, technology adoption, and trust in AI systems may vary across cultural and institutional contexts, the observed relationships may differ in other regions or educational systems. Future studies could replicate the model in different countries or educational levels to examine whether the relationships remain consistent across diverse learning environments.

Third, several methodological and measurement-related limitations should be acknowledged. The study relied on self-reported measures across all constructs and employed a single-time-point cross-sectional design, which may introduce concerns related to common method bias, subjective evaluation, and limitations in establishing causal relationships. In particular, the use of perceived English proficiency rather than objective performance measures may influence the strength of the observed relationships. Although all constructs satisfied the minimum recommended thresholds for reliability and convergent validity, the AVE values for AI-assisted task-based language learning and self-regulated learning remained at the threshold level (AVE = 0.500), and a small number of item loadings fell below the preferred 0.70 criterion. Sensitivity analyses indicated that removing lower-loading items produced only marginal improvements in measurement quality without materially altering the structural relationships; therefore, the items were retained to preserve conceptual comprehensiveness and content validity. Nevertheless, these findings suggest that the measurement properties of the AI-assisted task-based language learning construct should be interpreted with appropriate caution and further refined in future studies. Future research may incorporate objective indicators of language proficiency, longitudinal or experimental research designs, and more context-specific measurement instruments to better capture the complexity of AI-assisted language learning environments.

Fourth, although the study examined cognitive engagement and self-regulated learning as mediating mechanisms, other potential psychological processes such as learning satisfaction, perceived usefulness of AI tools, or digital learning motivation may also influence the relationship between AI-assisted learning and language outcomes. This is especially relevant given the relatively lower explanatory power observed for cognitive engagement, suggesting that additional factors may contribute to learners’ engagement in AI-supported environments. Future research could extend the model by incorporating these additional mechanisms.

Fifth, while AI trust was examined as a moderator, future studies may explore other boundary conditions, such as digital literacy, technological readiness, or instructor support, to further understand the conditions under which AI-assisted learning environments become most effective. Considering the relatively modest moderating effect observed in this study, examining additional contextual factors may provide a more comprehensive understanding of how and when AI-assisted learning produces optimal outcomes.

Finally, future studies may additionally employ Importance–Performance Map Analysis (IPMA) to identify which instructional or psychological constructs deserve greater practical attention in AI-assisted language learning environments.

## Conclusion

In conclusion, this study examined how AI-assisted task-based language learning influences English proficiency among university students in China by investigating the mediating roles of cognitive engagement and self-regulated learning, as well as the moderating role of AI trust. The findings demonstrate that AI-supported task-based learning environments significantly enhance learners’ cognitive involvement and self-regulatory learning behaviors, which in turn contribute to improved English proficiency. Notably, the results suggest that AI-assisted learning does not operate through direct instructional effects alone, but primarily through underlying cognitive and behavioral mechanisms that shape how learners engage with and regulate their learning processes. The results further indicate that students’ trust in AI systems strengthens the effectiveness of AI-assisted learning approaches, highlighting the importance of psychological perceptions of technology in educational contexts. However, trust functions as a supporting boundary condition rather than a dominant driver, emphasizing that effective instructional design and learner processes remain central to learning outcomes. By integrating instructional practices, learner cognitive processes, and technology-related trust within a unified framework, the study provides a more comprehensive understanding of how AI-enabled learning environments can support language learning outcomes in higher education. Overall, the study offers a mechanism-based perspective on AI-assisted language learning, contributing to a more nuanced understanding of how technology, learner behavior, and psychological perceptions jointly shape educational effectiveness.

## Data Availability

The raw data supporting the conclusions of this article will be made available by the authors, without undue reservation.
