# Supplementary material for: AI-assisted task-based language learning and English proficiency: a dual-pathway model of cognitive engagement and self-regulated learning with AI trust as a boundary condition
Source: Front Psychol. 2026 Jun 15;17:1842773. doi: 10.3389/fpsyg.2026.1842773 (PMC13312160; doi:10.3389/fpsyg.2026.1842773)
Supplement: Supplementary file 1 [file Supplementary_file_1.docx]

**Appendix A: Measurement Items**

All items were measured using a five-point Likert scale (1 = strongly disagree, 5 = strongly agree).

**AI-Assisted Task-Based Language Learning**

AIL1: AI-supported tasks help me practice English in realistic communication situations.

AIL2: I use AI tools to complete English learning tasks effectively.

AIL3: AI-assisted activities improve my participation in English learning tasks.

AIL4: AI-supported learning helps me apply English in practical situations.

AIL5: I actively engage with AI tools during English task-based activities.

AIL6: AI-supported tasks provide useful feedback for improving my English.

AIL7: AI-assisted learning makes English tasks more interactive.

AIL8: AI tools help me complete English learning tasks more efficiently.

**Cognitive Engagement**

CE1: I try to understand the underlying meaning of English tasks rather than just completing them.

CE2: I put a lot of effort into understanding English learning tasks.

CE3: I think deeply about the content when completing English tasks.

CE4: I try to connect new English knowledge with what I already know.

CE5: I persist even when English tasks are challenging.

**Self-Regulated Learning**

SRL1: I set goals for improving my English when completing learning tasks.

SRL2: I monitor my progress when learning English.

SRL3: I adjust my learning strategies when I do not understand something.

SRL4: I plan my learning activities when working on English tasks.

SRL5: I evaluate my performance after completing English tasks.

**English Proficiency**

EP1: I can effectively express my ideas in English during academic activities.

EP2: I can understand English materials used in my courses.

EP3: I can communicate clearly in English in classroom discussions.

EP4: I feel confident in using English for academic purposes.

EP5: My overall English proficiency is strong.

**AI Trust**

AIT1: I trust the suggestions provided by AI tools when learning English.

AIT2: AI tools provide reliable support for my English learning.

AIT3: I feel confident using AI tools for learning English.

AIT4: AI systems provide useful recommendations for improving my English.

AIT5: I believe AI tools enhance my English learning experience.

| Appendix B. Means, standard deviations, and correlations | | | | | | | |
| --- | --- | --- | --- | --- | --- | --- | --- |
| Constructs | Mean | SD | 1 | 2 | 3 | 4 | 5 |
| 1. AI-assisted task-based language learning | 3.71 | 0.62 | — |  |  |  |  |
| 2. AI trust | 3.68 | 0.66 | .66** | — |  |  |  |
| 3. Cognitive engagement | 3.59 | 0.59 | .30** | .36** | — |  |  |
| 4. English proficiency | 3.64 | 0.61 | .62** | .70** | .42** | — |  |
| 5. Self-regulated learning | 3.73 | 0.57 | .45** | .50** | .29** | .63** | — |
